# Tuning the F695 fluorescent state in photosystem II using site-directed mutagenesis in *Synechocystis sp.* PCC 6803

**DOI:** 10.1007/s11120-026-01217-1

**Published:** 2026-05-18

**Authors:** Amala Phadkule, Amit Srivastava, Alexandria Alailima Martin, Lauren G. Dome, Steven D. McKenzie, Sujith Puthiyaveetil, Mike Reppert

**Affiliations:** 1 James Tarpo Jr and Margaret Tarpo Department of Chemistry, Purdue University, 560 Oval Drive, West Lafayette, IN 47907 USA; 2 Department of Biology, Saint Louis University, St. Louis, MO 63103 USA; 3 Department of Biochemistry, Purdue University, West Lafayette, IN 47907 USA; 4 Center for Plant Biology, Purdue University, West Lafayette, Indiana, 47907 USA

**Keywords:** *Synechocystis sp*, F695, Photosystem II, PSI-knockdown, 77 K fluorescence

## Abstract

In photosynthetic proteins, pigments at a higher energy level funnel excitation energy to pigments at a lower energy level. Specifically, in Photosystem II (PSII), energy is transferred downhill to the reaction center (RC), where water splitting occurs. However, the lowest-energy state in PSII is not the RC, but the F695 state, which can be observed using low-temperature spectroscopy. This lowest-energy state is typically assigned to a monomeric pigment, Chl B16 (ligated by His114), but this assignment has been called into question based on theoretical fits to low-temperature spectra. In this study, we set out to test concretely whether the F695 state is localized on Chl B16 using site-directed mutagenesis and 77 K fluorescence spectroscopy. To reduce spectral congestion for whole-cell PSII studies, we developed a background strain (PSI-kd/$\Delta$PBS) that combines a Photosystem I (PSI) knockdown with a Phycobilisome (PBS) knockout. In this background strain, we made site-directed mutations at site Thr5 in the PsbH subunit, which forms a hydrogen bond with the $13^{1}$-keto group of Chl B16. All mutants were capable of heterotrophic growth (without noticeable differences from wild-type), indicating the PSII function remains intact. As expected for Chl B16-localized fluorescence, the Thr5$\rightarrow$Arg mutation red-shifted the F695 state due to the strengthening of the hydrogen bond, while the Thr5$\rightarrow$Ala mutation exhibits a blue shift as the hydrogen bond is eliminated. Taken together, these findings provide strong confirmation that Chl B16 is responsible for the lowest-energy state.

**Supplementary Information:**

The online version contains supplementary material available at 10.1007/s11120-026-01217-1.

## Graphical abstract

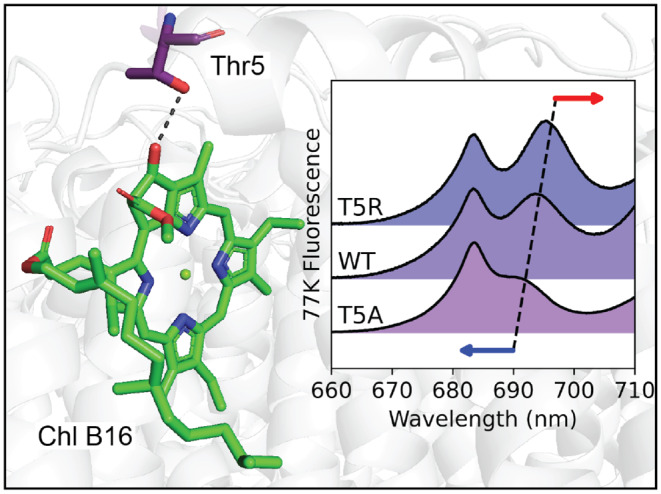

## Introduction

Photosynthetic organisms like plants, algae, and cyanobacteria perform oxygenic photosynthesis to convert absorbed solar energy into chemical energy. However, under natural conditions, less than 1% of the incident light energy is stored as biomass (Blankenship et al. 2011; Zhu et al. 2010; Walker 2009). Because evolution prioritizes reproductive success, photosynthetic organisms are optimized to capture light and nutrients rather than to maximize energy conversion efficiency (Anten 2004; Gust et al. 2008; Ort et al. 2015). As a result, a substantial fraction of absorbed light energy exceeds the capacity of the photosynthetic apparatus and is dissipated as heat, thereby limiting overall efficiency (Blankenship 2002; Green and Parson 2003; Melis 2009).

One approach to increase photosynthetic efficiency is to truncate the light-harvesting antenna  (Melis 2009; Ort et al. 2011). Removal of antenna proteins reduces the number of associated pigments, thereby decreasing excessive light absorption and minimizing energy loss through dissipation. This strategy has been successfully applied in some plants, algae, and cyanobacteria where antenna truncation was shown to improve the efficiency of light utilization for photosynthesis (Melis 2009; Ort et al. 2011; Neidhardt et al. 1998; Nakajima and Ueda 1999; Nakajima et al. 1998; Melis et al. 1998; Polle et al. 2003; Mussgnug et al. 2007; Mitra and Melis 2008; Sengupta et al. 2023; Beckmann et al. 2009; Shin et al. 2016; Kirst et al. 2014). However, in some cases in the cyanobacterium *Synechocystis sp.* PCC 6803 (referred to as S6803 hereafter) (Page et al. 2012; Liberton et al. 2013; Luimstra et al. 2019; Lea-Smith et al. 2014) and a study in *Chlamydomonas reinhardtii*  (de Mooij et al. 2015), antenna reduction led to a decrease in photosynthetic efficiency.

One potential difficulty with increasing photosynthetic efficiency through gene knockout is that the loss of entire protein assemblies could itself create functional challenges for living organisms.  (Liberton et al. 2017) In principle, more precise control over light-harvesting properties could be achieved by using site-directed mutagenesis to tune individual pigments within the target protein. For this approach to succeed, however, it is necessary to know the roles of individual pigments in excitation-energy transfer in the wildtype organism. Unfortunately, although many general design features of energy transfer in photosynthesis are well established (e.g., the “energy funnel”  (Blankenship 2002; Mančal 2013; Tiwari et al. 2013; Renger and Marcus 2002; Cheng and Fleming 2009; Ishizaki and Fleming 2009) whereby blue-to-red energy transfer between pigments concentrates energy near the reaction center (RC)), the specific optical properties and functional roles of individual pigments outside the reaction center are in most cases unknown.

Among the pigment-protein complexes found in nature, photosystem II (PSII) is of particular interest because it catalyzes the water-splitting reaction that initiates oxygenic photosynthesis. Within PSII, the core antenna complexes CP43 and CP47 bind 13 and 16 chlorophylls, respectively, and serve to funnel excitation energy to the RC from sunlight and peripheral antennas  (Vasil’ev et al. 2001). Surprisingly, however, 77 K fluorescence measurements reveal that the lowest-energy state in PSII is not in the RC but a 695 nm fluorescent state roughly 10 nm to the red from the RC and originating from the CP47 complex  (Dorssen et al. 1987; de Weerd et al. 2002). This energy state is conventionally termed as “F695” although the peak position changes slightly ($\pm 1$ nm) with temperature and varies somewhat from sample to sample in purified preparations (Neupane et al. 2010; Acharya et al. 2010; Kalaji et al. 2017). The F695 state is speculated to have a photoprotective function, (Reppert et al. 2010; Shibata et al. 2013; Reimers et al. 2016) although uncertainty in the structural assignment of the state to a specific pigment within CP47 makes detailed functional analysis difficult.

**Fig. 1:**
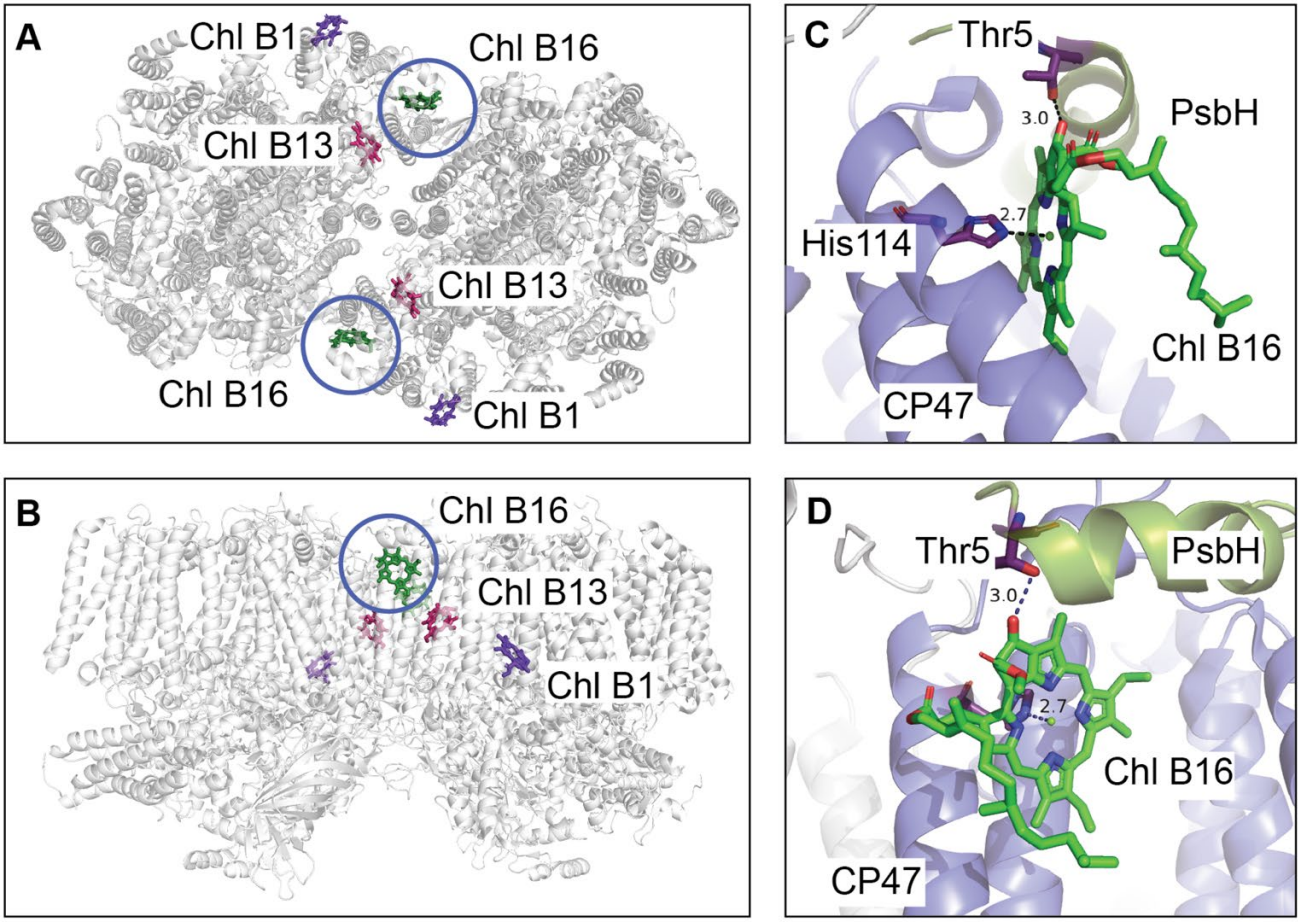
Structural overview of Chl B16 and its environment in PSII. PSII structure based on 7N8O  (Gisriel et al. 2021). (**A**) top view of the PSII dimer highlighting chlorophylls proposed as the lowest-energy state: traditional assignment Chl B16 (green, circled), and alternative candidates Chl B1 (violet) and Chl B13 (magenta). (**B**) side view showing the same chlorophylls. (**C**) detailed view of Chl B16 coordination by His114 (CP47 subunit), which ligates the $\textrm{Mg}^{2+}$ center. (**D**) detailed view of the hydrogen bond between Thr5 (PsbH subunit) and the $13^{1}$-keto group of Chl B16

In a seminal CP47 mutagenesis study, Shen and Vermaas observed the loss of the 695 nm peak when the His114 or His23 residues were mutated to Tyr  (Shen and Vermaas 1994). When His114 was replaced with Gln, the authors observed a blue shift of $\approx 2$ nm in the 695 nm peak, while the mutation of His23 to Asn resulted in a decrease in the amplitude of the 695 nm peak. The authors suggested that mutation of the conserved His residues results in the loss of $\textrm{Mg}^{2+}$ in chlorophyll due to the absence of its fifth ligand. This study was the first to suggest that the chlorophyll Chl 627 (Chl B16, standard numbering is compared with traditional numbering for CP47 chlorophylls in Table 1) ligated by the His114 and/or Chl 623 (Chl B12) ligated by His23 played an important role in the stable assembly of PSII. In a CP43 mutagenesis study that followed, Manna and Vermaas concluded that the Chl 627 (Chl B16) bound to His114 is associated with the lowest-energy state in PSII  (Manna and Vermaas 1997) (Fig. 1A, B).

**Table 1:** Correspondence between chlorophyll numbering schemes in PSII. Comparison of chlorophyll nomenclature across PSII structures 2AXT  (Loll et al. 2005), 3BZ1  (Guskov et al. 2009) (this structure is superseded by 4V62), 3ARC  (Umena et al. 2011) (this structure is superseded by 3WU2), and 7N8O  (Gisriel et al. 2021) with the standard numbering scheme  (Müh and Zouni 2020) adopted throughout this study

2AXT (Loll et al. 2005)	3BZ1 (Guskov et al. 2009)	3ARC (Umena et al. 2011)	7N8O (Gisriel et al. 2021)	Standard numbering (Müh and Zouni 2020)
11	511	612	601	B1
12	512	613	602	B2
13	513	614	603	B3
14	514	615	604	B4
15	515	616	605	B5
16	516	617	606	B6
17	517	618	607	B7
21	518	619	608	B8
22	519	620	609	B9
23	520	621	610	B10
24	521	622	611	B11
25	522	623	612	B12
26	523	624	613	B13
27	524	625	614	B14
28	525	626	615	B15
29	526	627	616	B16

This assignment was supported by a subsequent 77 K absorption and linear dichroism study of CP47 complexes, by de Weerd et al., which concluded that the oscillator strength for the F695 state corresponded to $\approx$1 chlorophyll  (de Weerd et al. 2002). This result was further strengthened by Andrizhiyevskaya et al. in a study that explored the origin of the 77 K F685 and F695 bands in PSII  (Andrizhiyevskaya et al. 2005). A modeling study on the PSII core complexes calculated site energies for pigments in CP47 and concluded that Chl B16 has the lowest site energy  (Raszewski and Renger 2008).

However, some simulation studies have called this assignment into question. A study of simulated steady-state absorption, emission, and nonresonant hole burning spectral data based on experimental results of CP47 antenna complex suggested that Chl 523 (Chl B13) may strongly contribute to the lowest-energy state  (Reppert et al. 2010) (Fig. 1A, B). Conversely, Shibata et al. compared experimental time-resolved fluorescence spectroscopy data with simulations that supported the traditional assignment to Chl 526 (Chl B16)  (Shibata et al. 2013). Two more modeling studies indicated that it is Chl 523 (Chl B13) or Chl 526 (Chl B16) that is the origin of the F695 state  (Reinot et al. 2016; Jassas et al. 2017). Meanwhile, liquid-helium temperature parallel circular dichroism and circularly polarized luminescence measurements suggested a new candidate, Chl 612 (Chl B1), as the lowest-energy state  (Hall et al. 2016) (Fig. 1A, B). Finally, another mutagenesis study by D’Haene et al. supported the most common assignment of Chl 627 (Chl B16) by showing that deletion of the PsbH subunit (which interacts with Chl B16 through a pigment-protein hydrogen bond) resulted in the loss of the 695 nm peak  (D’haene et al. 2015).

One potential source of such discrepancies is the different methods of sample preparation of the CP47 complex, since even small variations in sample preparation can produce substantial shifts in F695 fluorescence (Neupane et al. 2010; Acharya et al. 2010; Kalaji et al. 2017). A second point of contention is whether the spectroscopic changes observed in mutagenesis studies might be explained by non-specific structural perturbations of the entire complex (and thereby the site energies of other pigments), rather than local changes at site B16. Although the reported mutations specifically target the B16 site (either through mutation of the His residue that ligates Chl B16 or deletion of the adjacent PsbH subunit), nonspecific structural effects are difficult to exclude entirely  (Reppert et al. 2010; Reinot et al. 2016; Jassas et al. 2017; Hall et al. 2016).

**Fig. 2:**
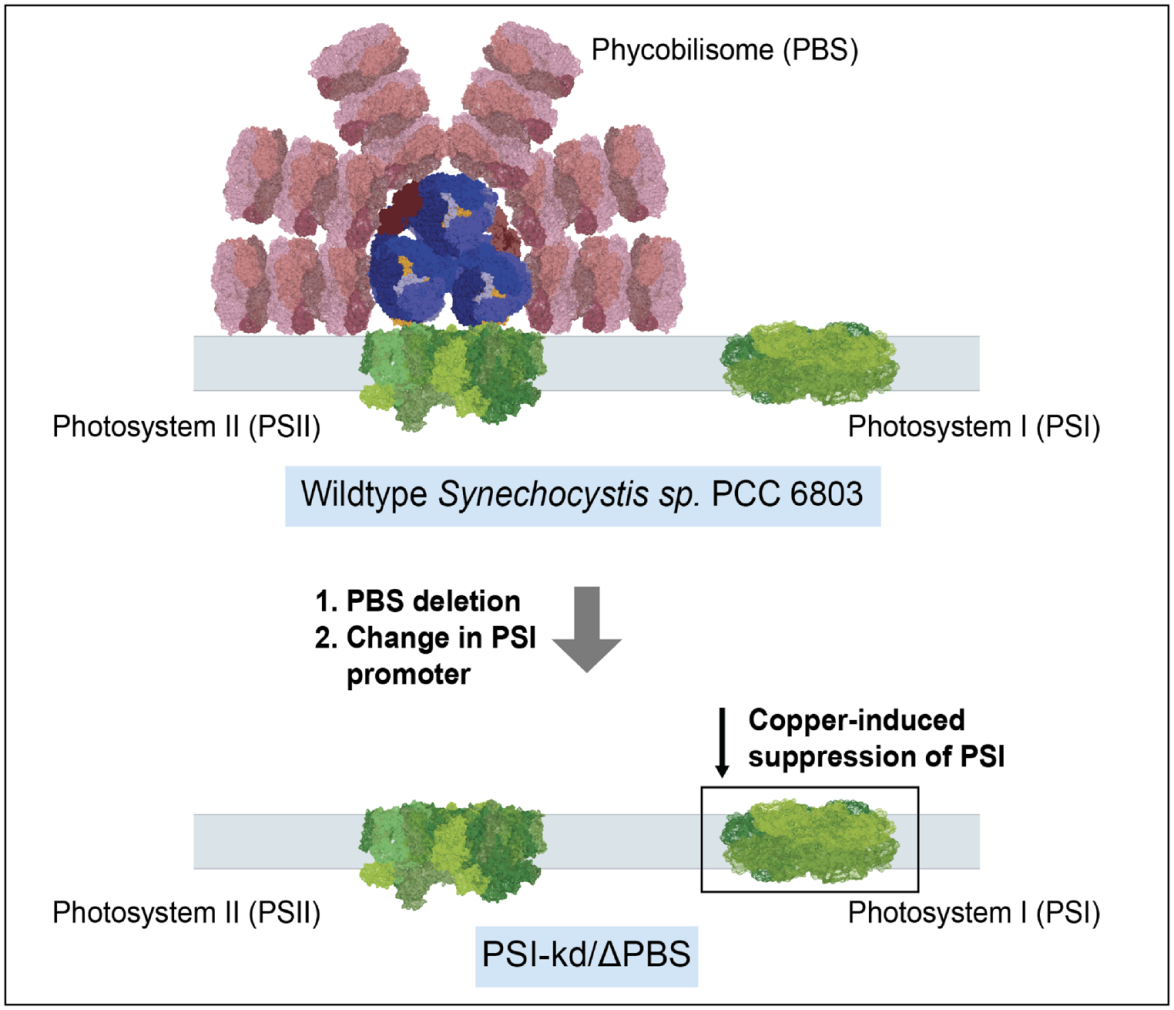
Generation of PSI-kd/${\rm{\Delta }}$PBS through mutagenesis. Schematic showing the transformation of wildtype S6803 to the background strain PSI-kd/$\Delta$PBS through two steps: 1) deletion of PBS 2) replacing the PSI promoter to a copper-repressible promoter *PpetJ*

The motivation for this study is to provide an orthogonal and minimally perturbative test of the assignment of the F695 state to Chl B16 through site-directed mutations that modify hydrogen-bonding to the B16 pigment without modifying its native ligand or deleting nearby subunits (Fig. 1C, D). To improve spectroscopic clarity (without the need for potentially destabilizing isolation of the complex), we developed a background strain (PSI-kd/$\Delta$PBS) in S6803 that combines phycobilisome (PBS) deletion with inducible Photosystem I (PSI) knockdown, eliminating interfering fluorescence signals and enabling clearer visualization of PSII-specific spectral features (Fig. 2).

Our results provide strong evidence for the assignment of the F695 state to Chl B16, as originally suggested by Shen and Vermaas  (Shen and Vermaas 1994) and supported by the more recent work of D’Haene et al.  (D’haene et al. 2015) With the exception of the conservative T5S mutation, all seven Thr5 mutants constructed exhibit notable changes to the F695 band, either in the form of frequency shifts or lost fluorescence signal. Most importantly, of the three mutants with clearly resolved F695 signal – T5A, T5S, and T5R – the observed frequency shifts agree with the trends expected from simple electrostatics: eliminating the hydrogen bond (T5A) induces $\sim$ 2 nm blue-shift, while strengthening the hydrogen bond (T5R) induces $\sim$ 1.5 nm redshift  (de Weerd et al. 2002; Krawczyk 1989; Braun et al. 2003; Silber et al. 2008; Romero et al. 2012; Llansola-Portoles et al. 2020; Srivastava et al. 2021; Wang et al. 2025). Conversely, the conservative T5S mutation induces modest broadening of the F695 peak without changing the peak frequency. These findings also confirm the recent suggestion that modifications to Thr5 hydrogen bonding observed in certain far-red PSII structures should induce a blueshift in the Chl B16 electronic transition  (Gisriel et al. 2023).

## Methods

### Cyanobacterial strains and culture conditions

Cyanobacterial strain *Synechocystis sp.* PCC 6803 was used as the wild type (WT) in this study. The derivative strains, a knockdown strain of PSI (PSI-kd) and a phycobilisome-less strain ($\Delta$PBS), were constructed from the WT (Fig. 2).

**Fig. 3:**
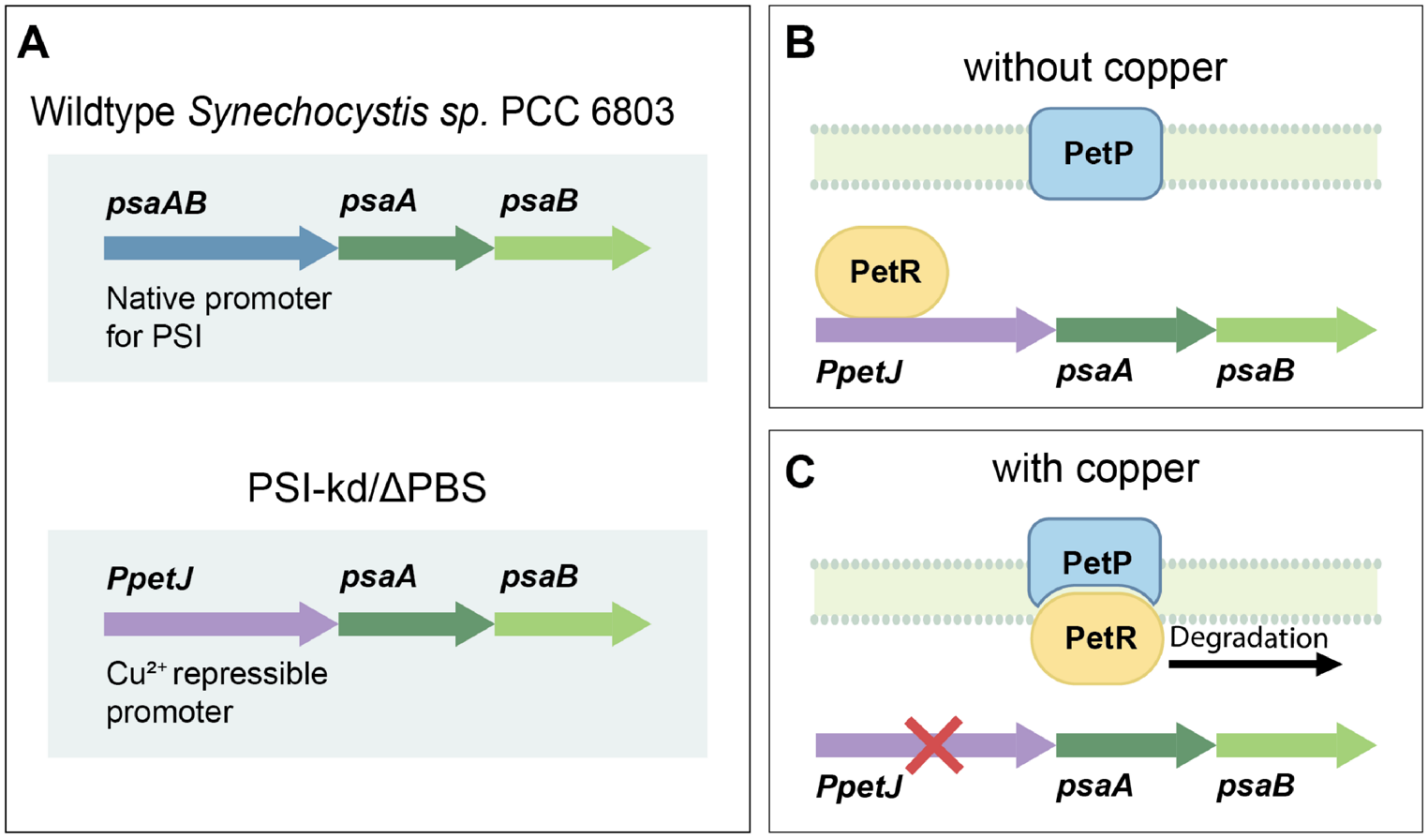
Generation and mechanism of PSI knockdown using *PpetJ*. (**A**) The native *psaAB* promoter is replaced with a copper-repressible promoter, *PpetJ*, enabling copper-dependent repression of PSI expression. (**B**) As described in Reference  (García-Cañas et al. 2021), without copper in the medium, the membrane protease, PetP, does not break down the transcription factor, PetR. PetR activates *PpetJ* and leads to protein expression. (**C**) with copper in the medium, PetP breaks down PetR through proteolysis. Without PetR to activate *PpetJ*, protein expression is suppressed.

The PSI-kd strain was generated by replacing the native promoter *psaAB* of the PSI genes *psaA* and *psaB* with a copper-repressible promoter, *PpetJ *(Fig. 3, Fig. S1). *PpetJ*, which natively regulates cytochrome $c_{6}$ expression in cyanobacteria, is repressed in the presence of copper through a mechanism detailed by García-Cañas et al.  (García-Cañas et al. 2021) and illustrated schematically in Fig. 3. Inspired by this and related work, (García-Cañas et al. 2021; Angeleri et al. 2019; Zhangs et al. 1992) we incorporated *PpetJ* for copper-dependent regulation to achieve controlled PSI expression. In the absence of copper, the transcription factor PetR activates *PpetJ*. But in the presence of copper, the protease PetP degrades PetR, resulting in reduced availability of PetR to activate *PpetJ*. The exact mechanism by which copper acts in the regulation of *PpetJ* by PetP and PetR is not well understood.

The $\Delta$PBS strain was constructed through sequential deletions of *cpcBD*, *apcAC*, and *apcE*, generating intermediate single and double deletion mutants (Fig. S2–S5). The *cpcBD* operon encoding phycocyanin, the *apcAC* operon encoding allophycocyanin, and the *apcE* gene encoding the linker protein were individually replaced with gentamicin, erythromycin, and spectinomycin resistance cassettes, respectively. The double mutant PSI-kd/$\Delta$PBS was obtained by introducing the PSI-knockdown mutation into the $\Delta$PBS background (Fig. 2).

Site-directed mutagenesis was carried out in the WT, PSI-kd, $\Delta$PBS, and PSI-kd/$\Delta$PBS backgrounds by substituting threonine at position 5 of the PsbH subunit with different amino acids (Fig. S6). In each background, we replaced Thr5 with seven different residues: alanine (Ala, A), aspartic acid (Asp, D), glutamic acid (Glu, E ), histidine (His, H), lysine (Lys, K), arginine (Arg, R), and serine (Ser, S). Modified *psbH* sequences were synthesized in the pUC57 backbone (Synbio Technologies) and used to transform the respective background strains. All site-directed mutants carried a kanamycin resistance cassette (Fig. S6).

### Growth conditions

Cyanobacterial strains were maintained on BG-11 plates (2% agar) supplemented with glucose (10 mM), TES-NaOH buffer (pH 8.2, 10 mM), sodium bicarbonate (5 mM), sodium thiosulfate (1 mM), and appropriate antibiotics (chloramphenicol, $25\,\mu\textrm{g mL}^{-1}$; gentamicin, $5\,\mu\textrm{g mL}^{-1}$; erythromycin, $25\,\mu\textrm{g mL}^{-1}$; spectinomycin, $25\,\mu\textrm{g mL}^{-1}$; kanamycin, $25\,\mu\textrm{g mL}^{-1}$). The plates were kept at $28^{\circ}\,\textrm{C}$ under $10\,\mu\textrm{E}$ light in an AlgaeTron growth chamber (Photon Systems Instruments). Liquid cultures were grown under the same conditions mixotrophically, supplemented with 5 mM glucose and 25 mM HEPES-NaOH buffer (pH 7.5).

### Copper-regulated PSI suppression

The PSI-kd and PSI-kd/$\Delta$PBS background strains, along with their site-directed mutants, were maintained on glucose-supplemented BG-11 plates without copper sulfate, thereby eliminating the sole copper source from the medium. Liquid cultures were similarly grown in glucose-supplemented, copper-free medium in Erlenmeyer flasks. Copper-deficient conditions have been shown to be non-stressful for S6803 growth, and concentrations up to 2 $\mu$M $\textrm{Cu}^{2+}$ do not adversely affect growth rates  (Giner-Lamia et al. 2014). Within this range, copper homeostasis mechanisms regulate the expression of electron transfer proteins such as plastocyanin (*petE*) and cytochrome $c_{6}$ (*petJ*), allowing precise control of the photosynthetic machinery without imposing cellular stress  (Angeleri et al. 2019; Zhangs et al. 1992).

**Fig. 4:**
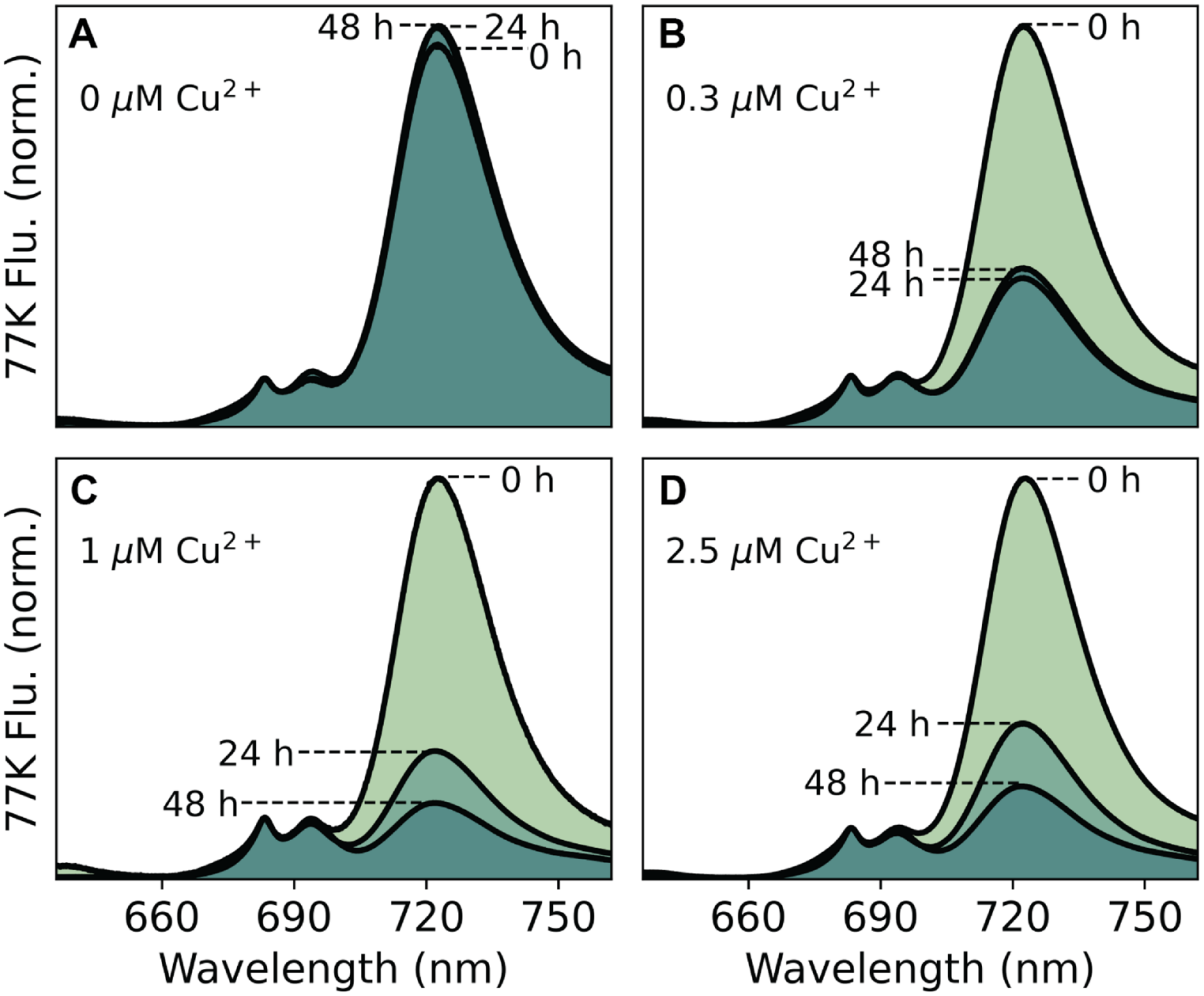
PSI suppression in PSI-kd/$\Delta$PBS under different copper concentrations. Time-course of PSI suppression monitored by 722 nm fluorescence emission at 77 K. (**A**) Without copper (0 $\mu$M $\textrm{Cu}^{2+}$), PSI levels increase over time. (**B**) Standard BG11 copper (0.3 $\mu$M) partially suppresses PSI after 48 h. (**C**) Increased concentration (1 $\mu$M $\textrm{Cu}^{2+}$) achieves improved suppression. (**D**) Higher concentration (2.5 $\mu$M $\textrm{Cu}^{2+}$) shows similar suppression to 1 $\mu$M but may approach cytotoxic levels   (Angeleri et al. 2019; Zhangs et al. 1992)

To perform PSI suppression experiments, strains were grown in copper-free medium to an $\textrm{OD}_{730}$ of 0.8–1.0, then diluted to $\textrm{OD}_{730} = 0.05 \pm 0.02$, which was designated as the zero-hour time point for fluorescence measurements. The degree of PSI suppression increased with higher copper concentration. Adequate suppression in the PSI-kd strain was achieved at $0.3\,\mu\textrm{M Cu}^{2+}$, whereas the PSI-kd/$\Delta$PBS strain required $1\,\mu\textrm{M Cu}^{2+}$ to achieve equivalent suppression (Fig. 4B, C, Table 2). The higher copper requirement for the PSI-kd/$\Delta\textrm{PBS}$ strain is likely attributable to the upregulation of the *petE* gene that encodes for plastocyanin, in the absence of PBS  (Liberton et al. 2017).

**Table 2:** Copper concentrations used to suppress PSI in PSI-kd/$\Delta$PBS. The degree of PSI suppression depends on the amount of copper in the growth medium.

Concentration of $\textrm{Cu}^{2+}$ used	Comments
0 $\mu$M	PSI suppression not observed
0.3 $\mu$M	Standard concentration of $\textrm{Cu}^{2+}$ in BG11 medium, suppresses PSI, but the degree of suppression does not decrease at 48 hrs
1 $\mu$M	Higher suppression compared to 0.3 $\mu$M $\textrm{Cu}^{2+}$
2.5 $\mu$M	Suppression level similar to 1 $\mu$M $\textrm{Cu}^{2+}$ but approach cytoxic copper levels (Giner-Lamia et al. 2014)

### Preparation of thylakoid membranes

Thylakoid membranes were isolated using the protocol described in References  (Komenda et al. 2019; Zakar et al. 2017) with some adaptation. Cultures (100 mL) were spun down at 7,000 rpm for 10 min, and the supernatant was discarded. The pellet was washed with 1.5 mL of buffer (25 mM MES/NaOH *p*H 6.5, $10\,\textrm{mM CaCl}_2$, $10\,\textrm{mM MgCl}_2$, 25% glycerol), then resuspended in 0.2 mL of the same buffer in a screw-cap tube. The same volume of glass beads was added to the tube, and the cells were lysed using a bead beater (Biospec Products, Mini-Beadbeater-16). Cells were lysed using five cycles of bead beating, and the tubes were chilled on ice for 2 min in between cycles. The lysate and unbroken cells were separated from the glass beads. To each tube, 0.2 mL buffer was added, followed by vortexing the tube for 10 s, and the liquid was transferred to a fresh tube. This process was repeated until all the material was transferred. The transferred mixture was spun down at $2500 \times\textrm{g}$ at $4^{\circ}\,\textrm{C}$ for 2 min. The unbroken cells and cell debris settled at the bottom, and thylakoids stayed in the buffer. The clear layer of thylakoids in buffer was transferred to a new 1.5 mL tube.

The thylakoids were spun down at 16,000 $\times$g for 20 min at $4^{\circ}\,\textrm{C}$. Pelleted thylakoids were resuspended in 0.2 mL of buffer. In a new 1.5 mL centrifuge tube, 10 $\mu$L of the thylakoids were added to 990 $\mu$L of methanol, then spun down at 16,000 $\times$g for 10 min. The supernatant was used to measure chlorophyll absorbance at 666 nm and 720 nm (Thermo Fisher Scientific, Nanodrop One). The following equation was used to calculate the chlorophyll concentration (Komenda et al. 2019; Zakar et al. 2017; Wellburn and Lichtenthaler 1984):

$$ [\text{Chl } (\mu \mathrm{g/mL})] = (A_{666} - A_{720}) \cdot 1269 \, \mu\mathrm{g/mL},$$

where $A_{666}$ and $A_{720}$ are optical density at 1 cm path length and wavelengths 666 nm and 720 nm, respectively.

### Clear native polyacrylamide gel electrophoresis

For sample preparation, 4 $\mu$g of thylakoid membranes were solubilized using 10% n-dodecyl $\beta$-D-maltoside to a final concentration of 0.8% maintaining the detergent to chlorophyll ratio at 25:1. The gel was run under conditions described in Refs.  (Komenda et al. 2019; Zakar et al. 2017). Fluorescence images were recorded for PSII dimer and monomer visualization, excited by Actinic 2 light ranging between 444 and 464 nm (Photon Systems Instruments, FluorCam 800-C/1010).

### 77 K fluorescence measurements

For spectroscopic measurements, 1 mL of culture was spun down for 1 min at room temperature at 5,000 rpm. The pelleted cells were resuspended in 40% glycerol to prevent crystal formation during 77 K measurements. A 10 $\mu$L plastic pipette tip (Fisherbrand, 02–707-454), transparent in the visible range, was used to hold the sample solution. The tip was secured with a wooden dowel and immersed in a liquid-nitrogen dewar (Horiba Instruments, FL-1013).

Samples were excited with a 450 nm continuous-wave diode laser (Thorlabs, CPS450) with a 1 mm spot size and 0.41 mW power at the sample position. The resulting fluorescence was analyzed with a spectrometer (Horiba Instruments, iHR320) equipped with a 600 grooves/mm diffraction grating and detected using a liquid-nitrogen-cooled CCD camera (Horiba Instruments, Symphony II), with a wavelength spacing of 0.126 nm between consecutive data points. All 77 K fluorescence spectra were corrected for the CCD spectral response. Fluorescence peak wavelengths were assessed simply as the wavelength of the pixel corresponding to maximum intensity after detector-response correction. Measurement error for assigning the fluorescence maximum was estimated for the WT F695 band in the PSI-kd/$\Delta$PBS background as the standard deviation $\sigma = 0.28$ nm across 19 collected fluorescence spectra obtained from 8 independent cultures collected on different days with varying degrees of PSI suppression. (The different spectra for each culture correspond to different time points from 24 to 72 hrs after initiating PSII suppression.) The corresponding standard deviations for the T5S and T5R mutants are $\sigma = 0.26$ nm and $\sigma = 0.18$ nm, as assessed in both cases from 9 spectra collected across 3 different growth cultures.

## Results

The conventional assignment for the origin of the F695 state is Chl B16, which is located at the stromal periphery of the thylakoid membrane in PSII (Fig. 1A, B). Chl B16 is ligated by the conserved residue His114 of the PsbB (CP47) subunit and interacts with its protein environment via a hydrogen bond (Fig. 1C, D) between its $13^{1}$-keto group and another conserved residue, Thr5 of the adjacent PsbH subunit. Previous mutagenesis studies targeting both of these interactions have implicated Chl B16 in 695 nm fluorescence, (Shen and Vermaas 1994; D’haene et al. 2015) but concerns have been raised as to whether the observed spectroscopic changes could be caused by more general structural perturbations to the complex, rather than specific changes at site B16 (Reppert et al. 2010; Reinot et al. 2016; Jassas et al. 2017; Hall et al. 2016).

To address this concern, we prepared a series of point mutations at the Thr5 site, designed to either break or strengthen the native Thr5 hydrogen bond. Hydrogen bonding to the $13^1$-keto has long been known to selectively tune Chl $\textrm{Q}_\mathrm{y}$ transition energies, (de Weerd et al. 2002; Krawczyk 1989; Braun et al. 2003; Silber et al. 2008; Romero et al. 2012; Llansola-Portoles et al. 2020; Srivastava et al. 2021; Wang et al. 2025) so the modification of such interactions via directed mutagenesis has proved an invaluable tool for probing the structural origins of individual spectroscopic features. (Krawczyk 1989; Braun et al. 2003; Silber et al. 2008; Romero et al. 2012; Llansola-Portoles et al. 2020; Srivastava et al. 2021; Wang et al. 2025; Ballschmiter and Katz 1969; Fowler et al. 1992; Remelli et al. 1999; Cogdell et al. 2002; Morosinotto et al. 2002; Kwa et al. 2004; Urboniene et al. 2007; Krausz 2013; Schlau-Cohen et al. 2014; Qian et al. 2021) Indeed, this strategy has already been applied to F695 fluorescence by D’Haene et al., (D’haene et al. 2015) who showed that deletion of the PsbH subunit (and thus the native Thr5 hydrogen bond) results in loss of the F695 band.

Our strategy of using site-directed mutagenesis (rather than subunit deletion) addresses in two ways the concern that larger-scale structural changes (rather than local changes at the B16 site) could explain the results of D’Haene et al. (D’haene et al. 2015) First, single point mutations are intrinsically less likely to destabilize protein complexes compared to subunit deletion, simply by virtue of the smaller structural changes involved. Second, the possibility to substitute several different amino acids in place of the WT Thr5 hydrogen-bond donor allows us to test whether any observed spectroscopic changes agree with known spectroscopic trends, namely that increased hydrogen-bond strength should introduce a fluorescence red shift, while decreased hydrogen bond strength (or hydrogen-bond elimination) should induce a blue shift. (Srivastava et al. 2021).

**Fig. 5:**
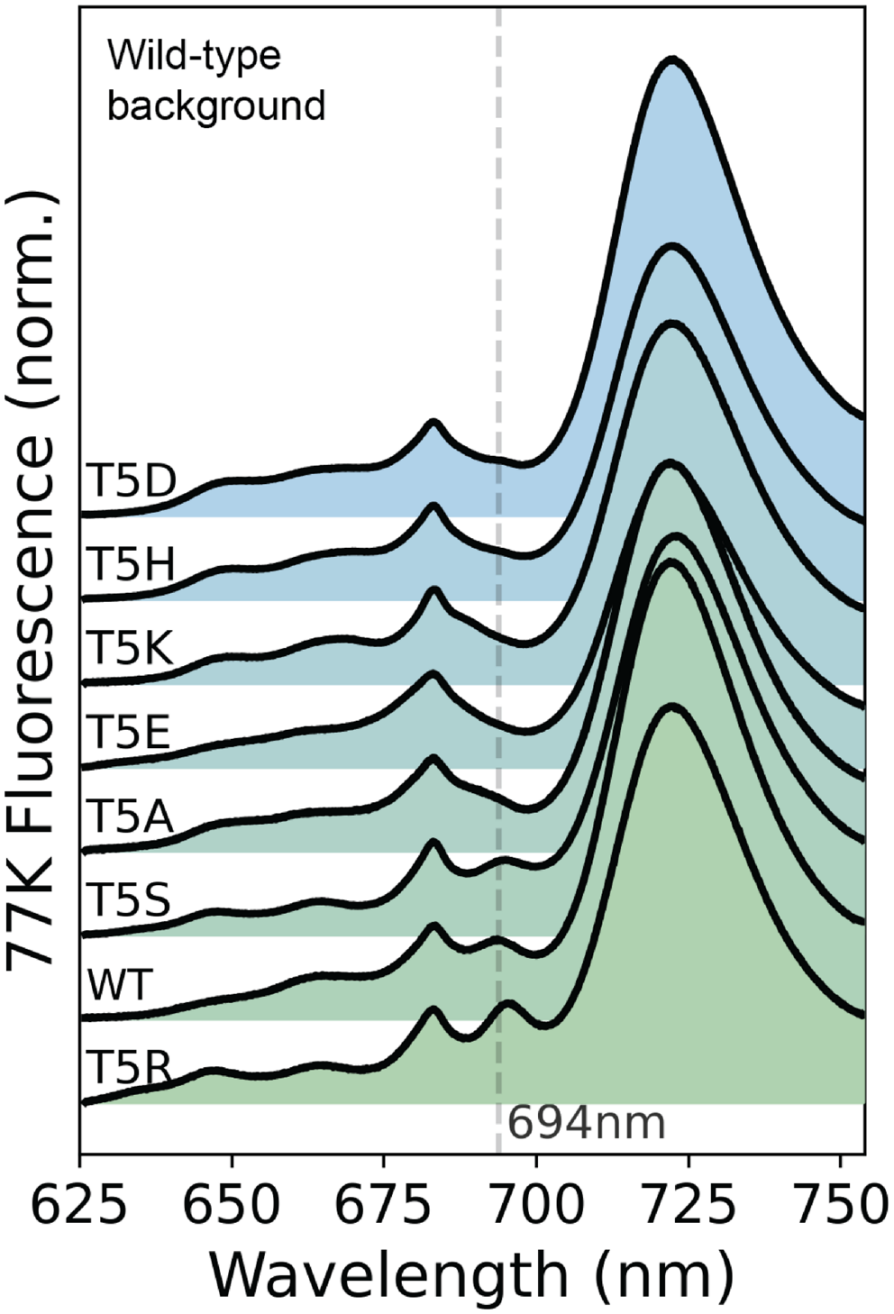
77 K emission spectra for Thr5 mutations in S6803. Fluorescence spectra of intact cells excited at 450 nm. The site-directed mutations at Thr5 (T5) include: T5R (*R* = Arg, a stronger H-bond donor), T5S (S = Ser, similar structure to WT), T5A (A = Ala, hydrophobic side chain eliminates H-bond), T5D/T5E (D = Asp and E = Glu, introduce a negative charge), T5K (K = Lys, expected to show stronger H-bond like Arg), T5H (H = His, introduces bulky side-chain) these mutations alter the 694 nm peak, but interpretation of these changes is complicated by spectral overlap from PSI (722 nm) and PBS (640–660 nm) emission bands

The 77 K fluorescence spectra (Fig. 5) for our Thr5 mutants in a WT S6803 background support these expectations, though the mutation-induced changes are obscured by fluorescence contributions from other complexes. Of the seven point mutations tested (replacing Thr5 with Ala (A), Arg(R), Ser(S), Lys(K), His(H), Asp(D), and Glu(E)), only two (T5S and T5R) exhibited F695 bands that remained clearly resolved from both the neighboring 685 nm peak due to the CP43 subunit and the stronger 722 nm band due to PSI. In the other five mutants, F695 intensity is decreased relative to WT, although in most cases a small shoulder appears on the red edge of the 685 nm CP43 band, presumably corresponding to blue-shifted F695 fluorescence. Importantly, all seven mutants grew well under both heterotrophic and autotrophic conditions (with rates comparable to WT), indicating that PSII function remains intact.

The observation of such pronounced spectroscopic changes in response to the (presumably local) perturbation of the Chl B16 hydrogen bond strongly supports the localization of the F695 state to the B16 pigment. Yet definite conclusions are made difficult by the obscurement of the modified F695 band by neighboring peaks. To circumvent this difficulty, we set out to prepare an S6803 strain with minimal spectroscopic interference from proteins other than PSII. The most important such contribution is a strong fluorescence band from PSI that peaks near 722 nm, with a weaker broad fluorescent background appearing between 650 and 660 nm due to various PBS subunits. Elimination of PSI fluorescence has previously been achieved in a PSI-less/${apcE}^-$ S6803 strain developed specifically as a background for fluorescence studies (Shen et al. 1993). Elimination of the various PBS bands has likewise been achieved in several studies, (Shen et al. 1993; Dzelzkalns and Bogorad 1986; Olive et al. 1997) typically focused on PBS function rather than spectroscopic analysis (Liberton et al. 2017; Plank et al. 1995; Rögner et al. 1990; Elmorjani et al. 1986; Ajlani and Vernotte 1998). However, no strain appears to have previously been generated that eliminates fluorescence contributions from both PBS and PSI components. While our immediate objectives here might be achievable in the existing PSI-less/${apcE}^-$ strain (Shen et al. 1993) (given the relatively weak fluorescence signal from the PBS), we wished to combine PBS and PSI suppression into a single background strain in anticipation of future absorption and hole-burning experiments, where overlap between PSII and the PBS is more problematic.

**Fig. 6:**
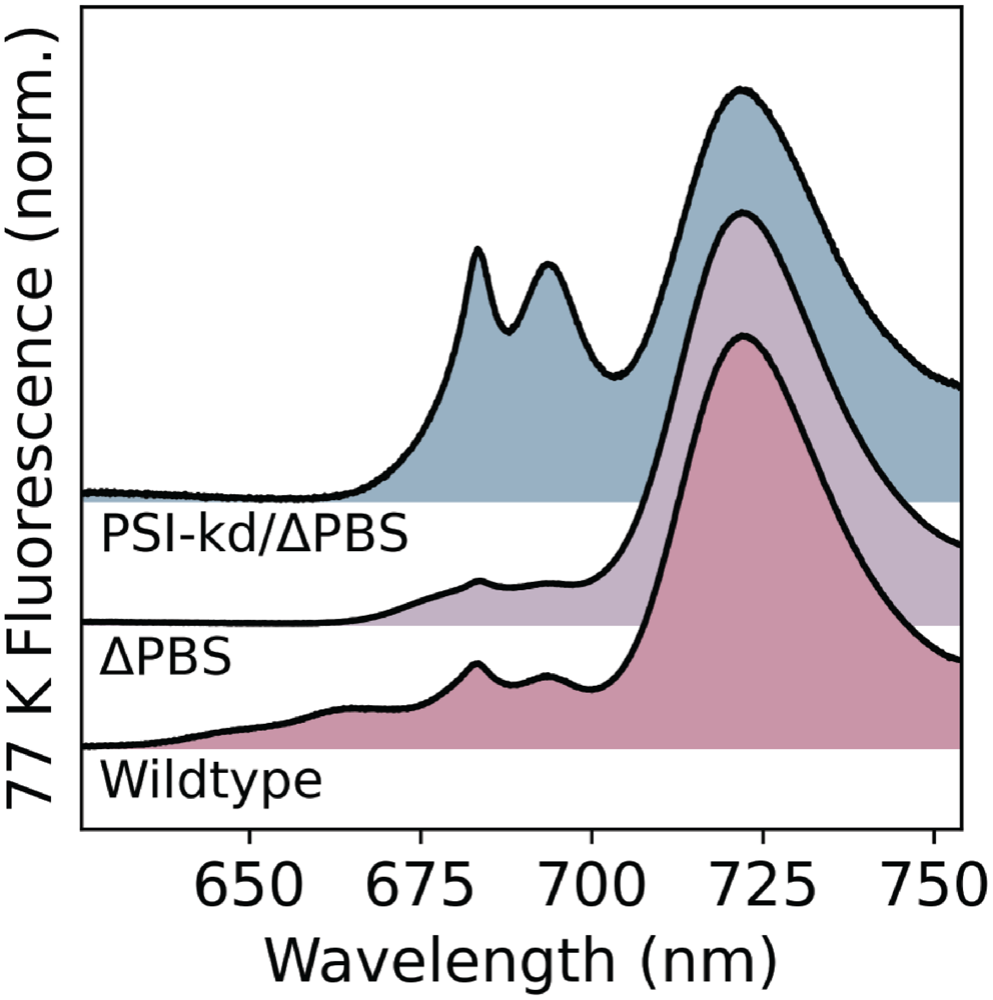
Comparison of 77 K fluorescence in background strains. 77 K fluorescence spectra comparing wildtype, $\Delta$PBS, and PSI-kd/$\Delta$PBS (after PSI suppression) background strains.

Our strategy for preparing this background strain is outlined schematically in Fig. 2. We first prepared a “$\Delta$PBS” strain lacking all major PBS components by deleting the operons encoding the phycocyanin rods, the allophycocyanin core, and the gene encoding the core linker protein (see Sect. “Methods”). This $\Delta$PBS strain grew well under the same light conditions as WT S6803, both with and without supplementation by glucose. (When growing for spectroscopic measurements, the strains were always supplemented with glucose to minimize variation in growth rates between mutants.) Whole-cell 77 K fluorescence spectra for this strain (Fig. 6) exhibit no signatures of PBS fluorescence in the 640–660 nm range, leaving a flat baseline on the high-frequency side of the twin PSII peaks at 685 and 695 nm. On the other hand, the intensity of PSI fluorescence increased relative to WT, with the intensity ratio of the 722 nm and 685 nm bands jumping from $\sim$5 in WT to nearly 10 in the $\Delta$PBS strain (Fig. 6).

Eliminating the PSI peak on the long-wavelength side of the PSII peaks proved to be more challenging, since our attempts to transform PSI knockout sequences into the $\Delta$PBS background failed to yield viable colonies. In response to this difficulty, we changed our strategy from entirely eliminating the PSI signal to reducing its intensity through gene knockdown. To this end, we replaced the native promoter of PSI (*psaAB*) with the copper-repressible promoter, *PpetJ* (Fig. 3). *PpetJ* (Angeleri et al. 2019; Zhangs et al. 1992) allows for PSI expression in the absence of copper and suppression of PSI production in the presence of copper. Although this strategy does not permit the complete elimination of PSI signal, we found that the degree of PSI suppression achievable in this manner was quite sufficient to unambiguously resolve the F695 band in our measurements.

The resulting PSI-kd/$\Delta$PBS strain showed no contribution from PBS fluorescence and reduced signal from PSI after copper-induced PSI knockdown (Fig. 6). For such measurements, the PSI-kd/$\Delta$PBS strain was grown in glucose-supplemented copper-deficient BG11, allowing for near-normal expression of PSI. During the log phase, $\textrm{Cu}^{2+}$ was added to suppress PSI production. After 72 h of growth in the presence of $\textrm{Cu}^{2+}$, the PSI fluorescence intensity is substantially reduced, with the ratio of the 685 nm and 722 nm bands dropping to $\sim$1.6, a three-fold reduction relative to WT S6803. In this background, both F685 and F695 PSII bands are clearly resolved from PSI emission at 77 K (Fig. 6).

**Fig. 7:**
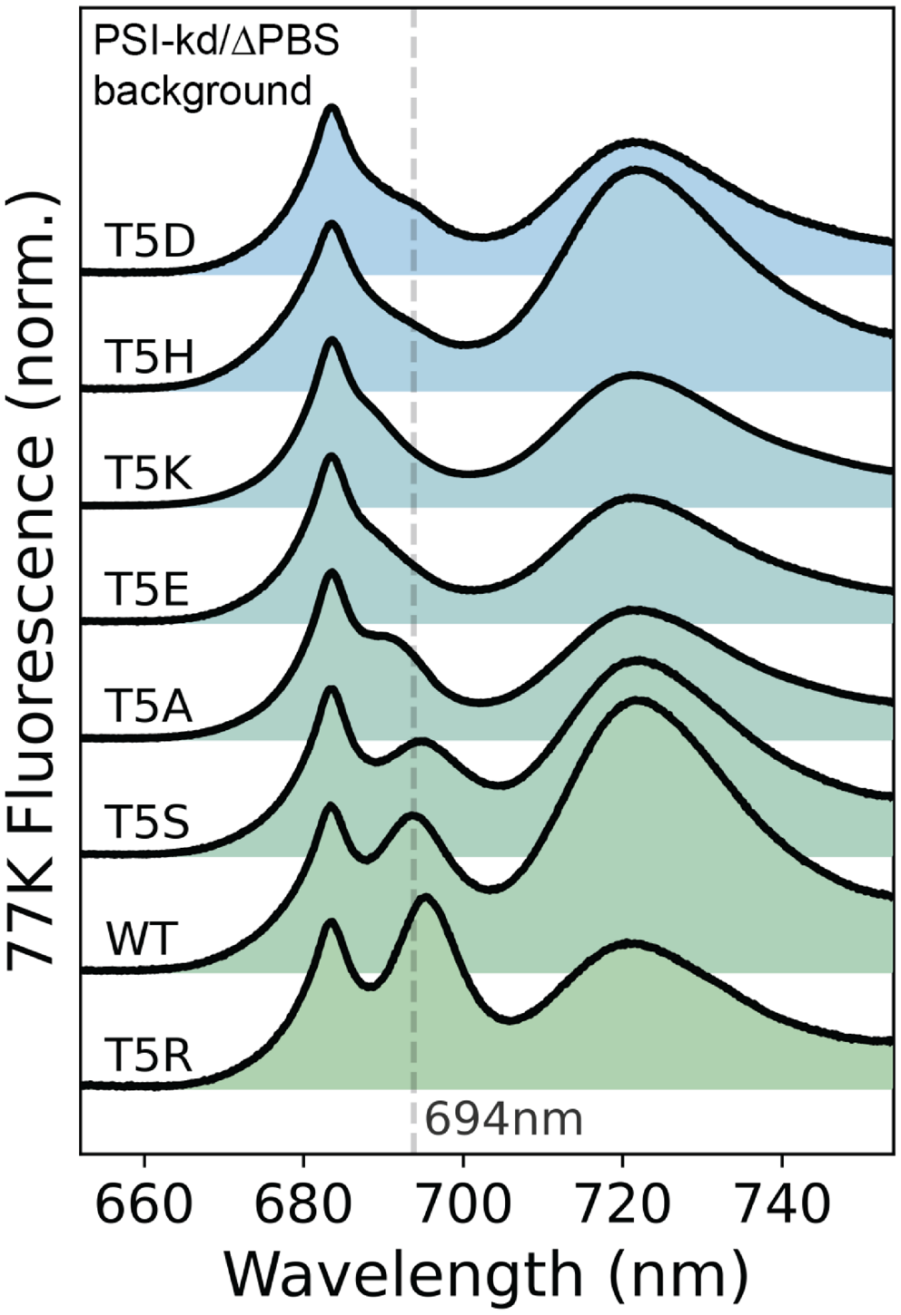
Thr5 mutations alter low-temperature fluorescence in PSI-kd/$\Delta$PBS. The shifts in F695 are easier to interpret in the PSI-kd/$\Delta$PBS background. The site-directed mutations at Thr5 (T5) show: T5R (*R* = Arg, stronger H-bond donor resulting in a red shift), T5S (S = Ser, similar structure to WT and shows no change), T5A (A = Ala, hydrophobic side chain eliminates H-bond and shows a blue shift), T5D/T5E (D = Asp and E = Glu, introduce a negative charge and are expected to induce a blue shift, but F695 is too weak to analyze reliably), T5K (K = Lys, expected to show stronger H-bond like Arg, but instead shows a weak possibly blue-shifted F695), T5H (H = His, introduces a bulky side-chain resulting in a weak F695 band.)

Finally, into this PSI-kd/$\Delta$PBS background strain, we transformed site-directed mutations at the PsbH-Thr5 site and collected 77 K emission spectra under PSI-suppression conditions (Fig. 7). Although fluorescent signals from PSI are still visible near 722 nm, the PSII bands are now easily distinguished, revealing the changes to the F695 state much more clearly. Compared to the WT protein, only the conservative T5S mutation leaves both the wavelength and intensity (relative to the F685 band) essentially intact, although even in this case, the F695 band appears slightly broadened. The T5R mutation exhibits a redshift from 693.86 $\pm$ 0.28 nm to 695.29 $\pm$ 0.18 nm (along with a small increase in fluorescence intensity), while T5A conversely shows a $\sim$ 2 nm blueshift to near 692 nm and a noticeable drop in intensity. For the remaining substitutions – Lys (T5K), His (T5H), Asp (T5D), and Glu (T5E) – the F695 band is no longer visible as a distinct band, though it is difficult to assess with certainty whether this is due primarily to a loss of intensity or a shift in wavelength. T5D appears more likely to reflect primarily a loss in intensity, since a faint shoulder is still visible at 694 nm; conversely, the T5E and T5K spectra are suggestive of a wavelength shift, since a very weak feature is visible near 690 nm. But in all four of these cases, the residual F695 band is too much obscured by the stronger F685 band to assign with confidence.

Finally, to check whether point mutations at the Thr5 site affect PSII dimer formation, we isolated thylakoid membranes from each Thr5 mutant in the PSI-kd/$\Delta$PBS background. Clear Native Polyacrylamide Gel Electrophoresis (CN-PAGE) results for the thylakoids (Fig. S7) show that mutants T5R, T5S, T5A, T5K, and T5D exhibit PSII dimer/monomer ratios comparable to the WT and PSI-kd/$\Delta$PBS strains. T5E shows a slight reduction in PSII dimer formation, while the T5H mutant appears almost entirely as a monomer in the CN-PAGE data. Thus, while PSII assembly appears unaffected in most of our Thr5 mutants, the T5H mutant appears to substantially perturb dimerization.

## Discussion and conclusions

The point-mutant spectra of Fig. 7 provide, in our view, definitive support for the traditional assignment of the F695 band to a localized transition on Chl B16 (ligated by His114 of CP47). The original motivation for this assignment comes from (Shen and Vermaas 1994), who reported that mutations at His114 result in loss and/or shifting of the F695 band. However, some subsequent simulation studies questioned whether these changes to the F695 band might be caused by a more general structural destabilization of the PSII complex in the His114 mutants, rather than the specific localization of the F695 state to Chl B16 (Reppert et al. 2010; Reinot et al. 2016; Jassas et al. 2017; Hall et al. 2016). To avoid this difficulty, the present study introduces point mutations at a different site, the Thr5 residue of PsbH, which in the WT protein forms a hydrogen bond with the $13^1-\textrm{keto}$ of chlorophyll B16. This strategy offers both an orthogonal test of the involvement of Chl B16 in F695 fluorescence and (since different protein side chains produce different hydrogen-bond interaction strengths) the possibility to rationally tune the F695 fluorescence wavelength based on well-established trends. (de Weerd et al. 2002; Krawczyk 1989; Braun et al. 2003; Silber et al. 2008; Romero et al. 2012; Llansola-Portoles et al. 2020; Srivastava et al. 2021; Wang et al. 2025) Relative to previous mutagenesis studies  (Shen and Vermaas 1994; D’haene et al. 2015) – which already provided strong support for the assignment of F695 to Chl B16 – our present results offer two useful safeguards against the concern that larger-scale structural changes could be responsible for observed F695 shifts  (Reppert et al. 2010;Reinot et al. 2016; Jassas et al. 2017; Hall et al. 2016).

First, the point mutations introduced here (which target a single hydrogen-bonding interaction) appear unlikely to produce large-scale structural changes that could affect fluorescence from pigments *other than* Chl B16. This expectation is supported by the observation that PSII dimer levels appears similar to WT for all mutants except T5H and to a lesser extent T5E. Moreover, the fact that all point mutants (in the WT background) grow normally under autotrophic conditions indicates, at any rate, that PSII function remains intact. Although not impossible, it is difficult to envision what sort of non-local change would lead to altered F695 fluorescence in all 6 non-conservative mutations without affecting functionality.

Second, the local character of the mutation-induced perturbation at Chl B16 is supported by the fact that the four samples with a discernible F695 band follow the physically expected trend in emission wavelength T5R > WT $\approx$ T5S > T5A. The linear correlation between $\textrm{Q}_\mathrm{y}$ transition frequency and the strength of hydrogen-bonding at the $13^1-\textrm{keto}$ group is well established both empirically and theoretically  (de Weerd et al. 2002;Krawczyk 1989; Braun et al. 2003; Silber et al. 2008; Romero et al. 2012; Llansola-Portoles et al. 2020; Srivastava et al. 2021; Wang et al. 2025). Physically, this trend has a simple explanation: strong hydrogen bonds (as presumably present in T5R) stabilize the electronic excited state, which has increased electron density in the vicinity of the $13^1-\textrm{keto}$ group relative to the electronic ground state  (Srivastava et al. 2021; Adolphs et al. 2008). This relative stabilization of the excited state lowers the HOMO - LUMO gap, yielding a lower transition frequency and absorption/emission at longer wavelengths. Conversely, breaking the native $13^1-\textrm{keto}$ hydrogen bond (as in T5A) destabilizes the electronic excited state and yields a shift to shorter wavelengths in fluorescence. Though it is of course possible that non-specific structural changes could happen to yield this same trend, this would seem a remarkable coincidence.

Though less obviously interpretable, it is interesting to note that the *intensity* of the F695 band follows the same trend (T5R > WT $\approx$ T5S > T5A) as the emission wavelength. Whether this reflects a change in the oscillator strength of the electronic transition, increased localization of energy at the B16 site, or simply tighter binding (and thus higher structural occupancy) of Chl B16, our data cannot distinguish. One possible route to answering this question would be time-resolved studies assessing both the oscillator strength of the Chl B16 transition and any variation in equilibration between states in response to Thr5 mutations.

This analysis raises the obvious question of what happens to F695 fluorescence in the remaining 4 mutants, representing substitution of Thr5 with K, H, D, and E. Naively, T5K would be expected to give similar results as T5R, while T5E and T5D should induce strong blue shifts (even more so than T5A) due to electrostatic repulsion between the negatively charged side chains and excited-state electron density.  (Srivastava et al. 2021) This description might plausibly fit T5E, but in all four cases the F695 band is too weak to be reliably interpreted. Though we cannot offer any definite conclusion, the simplest interpretation would seem to be that weakening of the F695 band corresponds simply to the partial elimination of Chl B16 from its native binding pocket. This appears particularly likely for the T5E and T5D mutants, where the negatively charged sidechains are likely to destabilize binding. The His sidechain of T5H is likewise much bulkier than the native Thr residue and may interfere sterically with pigment binding. The potential for structural destabilization in these mutants is supported by clear native polyacrylamide gel electrophoresis (CN-PAGE) experiments (Fig. S7), which reveal a noticeable decrease in PSII dimer/monomer ratio in the T5E and especially T5H mutants. From this perspective, T5K is the only mutant whose behavior appears really surprising in that it differs so strongly from the structurally similar T5R mutant yet shows strong dimer formation in CN-PAGE. The most likely explanation would seem to be that additional structural rearrangements (beyond simply modified hydrogen bonding) occur in the T5K mutant.

As to the question of why some simulation studies (including by one of the present authors  (Reppert et al. 2010)) found better agreement with low-temperature spectra when Chl B16 was assumed *not* to be the lowest-energy site, two factors are likely at play.  (Reppert et al. 2010; Reinot et al. 2016; Jassas et al. 2017; Hall et al. 2016) First, some degree of uncertainty in such simulations is unavoidable due both to the approximate nature of the available line shape theories and to the practical impossibility of testing all possible site-energy assignments. Second, and perhaps even more importantly in this case, the experimental spectra used in these studies to test and refine theoretical assignments are typically obtained from isolated CP47 complexes, which presumably lack the PsbH subunit. As previously shown by D’Haene et al., the interaction of PsbH with CP47 is essential to the formation of the intact F695 fluorescence band,  (D’haene et al. 2015), and our present results confirm the essential nature of the PsbH-Thr5 residue in particular. In this light, it is perhaps not surprising that simulation studies reliant on spectra from PsbH-deficient samples should fail to capture the essential role of Chl B16 in F695 fluorescence. Indeed, it is quite possible that 77 K fluorescence does *not* originate from Chl B16 in such samples, since loss of the PsbH subunit may perturb the B16 pigment sufficiently that (even if it is not lost during purification) it is no longer the lowest-energy pigment in the complex. Overall, this discussion highlights the importance of benchmarking both experimental and simulation work on isolated subunits against data for the intact core complex.  (Krausz et al. 2005)

In terms of the structural origins of Chl B16 redshift, our results support the suggestion by de Weerd et al.  (de Weerd et al. 2002) that the Thr5 hydrogen bond contributes to this shift, while at the same time highlighting that additional factors must also be involved. Although the trend from T5A to T5R confirms that strong hydrogen bonding tunes the Chl B16 transition to the red, the full range of tuning we observe in our experiments is only 4 nm (i.e., $\pm 2$ nm around WT). In comparison to the 10 nm redshift of the F695 band compared to the lowest-energy states of CP43 and 20 nm redshift compared to the average absorption frequency in PSII (around 675 nm  (Reppert et al. 2010)), this contribution appears rather modest. In this light, it is intriguing to note that Chl B16 is the only pigment in the CP47 complex that has a (psuedo) symmetry equivalent pigment in CP43 (Chl C13) but whose orientation is flipped by 180 degrees so that the His ligand approaches from the opposite side of the chlorin plane  (Lin et al. 2024). This difference in orientation produces a significant change in the doming deformation of the Chl B16 ring relative to its CP43 counterpart, though whether these changes are directly relevant to the redshifted transition frequency remains an open question  (Lin et al. 2024).

It is intriguing to note that the conserved Thr5 hydrogen bond is absent in the PSII structures of some WT organisms acclimated to far-red light, where Thr5 is natively replaced by A  (Gisriel et al. 2023). The fluorescence shift observed in our T5A mutant allows us to confirm that the suggestion of Gisriel et al. that this substitution produces a blue-shift of the Chl B16 transition, although (since other modifications to the binding pocket are also present in far-red PSII) the magnitude of the shift could be different from what we observe here.

Finally, we hope that the PSI-kd/$\Delta$PBS strain we introduce here may prove useful for future spectroscopic studies. Although only partial PSI suppression is possible with this strain, its fast growth rates (comparable to WT) under copper-deficient conditions makes it convenient to work with for applications where full PSI knockout is not required. In the future, it will be useful to explore whether even greater PSI suppression might be achieved in this strain under different lighting conditions  (Murakami 1997), a question we have not yet explored.

## Electronic supplementary material

Below is the link to the electronic supplementary material.

Supplementary Material 1

## Data Availability

All data supporting the findings of this study are available within the paper and its Supplementary Information or are available from the authors upon reasonable request.
