# Supplementary material for: Tuning the F695 fluorescent state in photosystem II using site-directed mutagenesis in Synechocystis sp. PCC 6803
Source: Photosynth Res. 2026 May 18;164(3):29. doi: 10.1007/s11120-026-01217-1 (PMC13183708; doi:10.1007/s11120-026-01217-1)
Supplement: Supplementary file 1 — Supplementary Material 1 [file 11120_2026_1217_MOESM1_ESM.pdf]

Supporting Information for Tuning the F695 Fluorescent State in Photosystem II Using Site-Directed Mutagenesis in *Synechocystis sp.* PCC 6803

Amala Phadkule[1], Amit Srivastava[2], Alexandria Alailima Martin[1], Lauren G. Dome[3], Steven D. McKenzie[3], Sujith Puthiyaveetil[3,4], Mike Reppert[1*]

[1*]James Tarpo Jr and Margaret Tarpo Department of Chemistry, Purdue University, 560 Oval Drive, West Lafayette, Indiana, 47907, USA.
[2]Department of Biology, Saint Louis University, St. Louis, 63103, Missouri, USA.
[3]Department of Biochemistry, Purdue University, West Lafayette, Indiana, 47907, USA.
[4]Center for Plant Biology, Purdue University, West Lafayette, Indiana 47907, USA.

*Corresponding author(s). E-mail(s): reppertm@purdue.edu; Contributing authors: aphadkul@purdue.edu; amit.srivastava@slu.edu; mart2098@purdue.edu; ldome@purdue.edu; smckenz@purdue.edu; spveetil@purdue.edu;

Construction of PSI knockdown

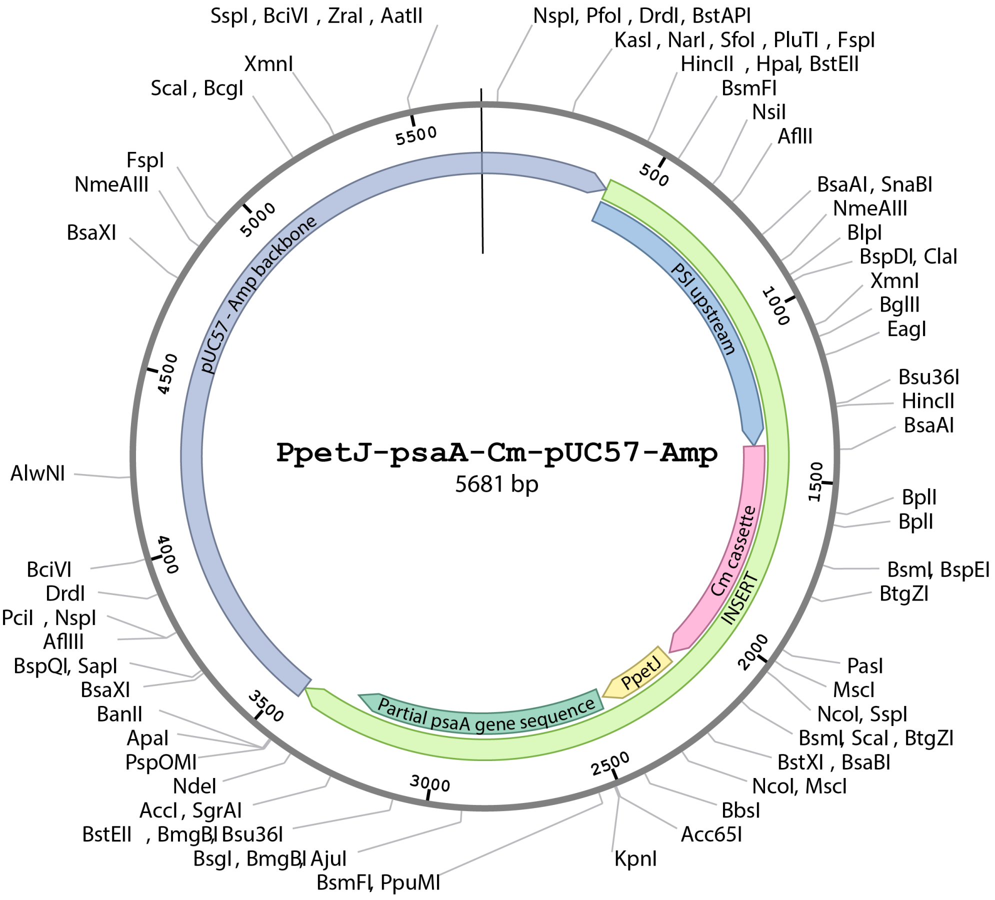

Fig. S1 Plasmid map of PpetJ-psaA-Cm-pUC57-Amp used to construct the PSI knockdown. Schematic representation of the pUC57 plasmid used to construct the PSI knockdown in WT S6803 and ΔPBS strains. Key features include the PSI upstream insert (1000 bp), chloramphenicol resistance cassette (772 bp), PpetJ promoter (280 bp), and partial psaA sequence (1000 bp). The plasmid was purchased from Synbio Technologies. The figure is generated using Benchling.

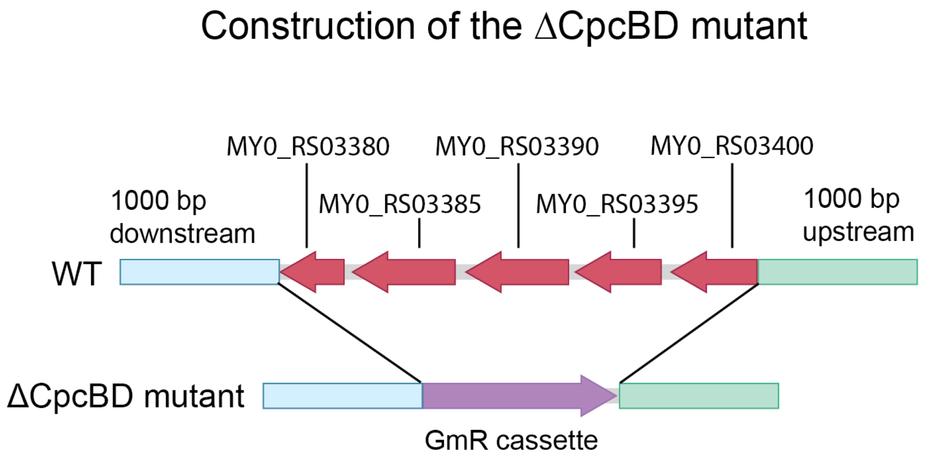

Fig. S2 Wildtype *Synechocystis sp.* PCC 6803 (WT) genes MY0_RSO3380, MY0_RSO3385, MY0_RSO3390, MY0_RSO3395, and MY0_RSO3400 are shown as red arrows. The upstream and downstream regions of 1000 bp each is highlighted on either side of the five genes. These genes and the bps in between them are all knocked out in the ΔCpcBD mutant and a Gentamicin resistance cassette (GmR) is inserted between the flanking regions.

Fig. S2 shows the method for the construction of the CpcBD deletion mutant.The genes MY0_RSO3380, MY0_RSO3385, MY0_RSO3390, MY0_RSO3395, and MY0_RSO3400 were knocked out. The transformation was made by using linear DNA that contained the Gentamicin resistance cassette flanked by 1000 bp on each side. The sequence for the flanking regions and the genes can be found on NCBI Reference sequence NC_020286.1 for *Synechocystis sp.* PCC 6803.

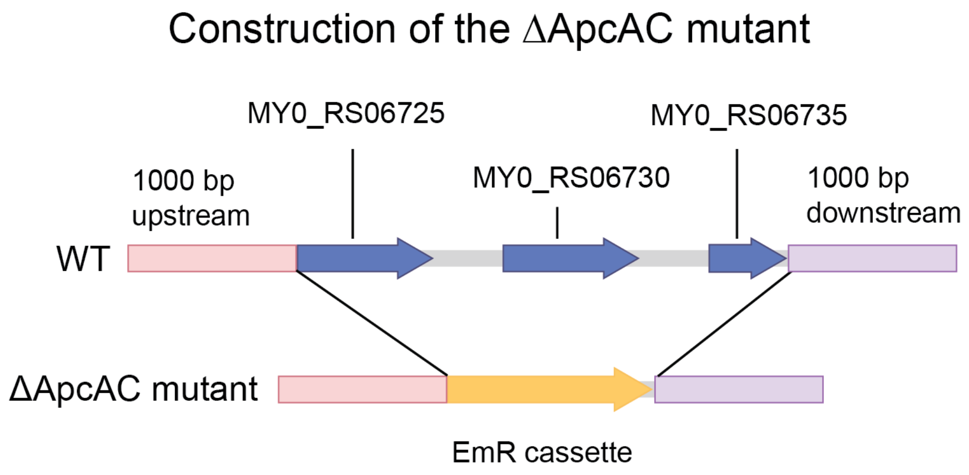

Fig. S3 Wildtype *Synechocystis sp.* PCC 6803 (WT) genes MY0_RSO6725, MY0_RSO6730, and MY0_RSO6735 are shown as blue arrows. The upstream and downstream regions of 1000 bp each is highlighted on either sides of the three genes. These genes and the bps in between them are all knocked out in the ΔApcAC mutant and a Erythromycin resistance cassette (EmR) is inserted between the flanking regions.

Fig. S3 shows the method used to construct the ApcAC deletion mutant. The three genes MY0_RSO6725, MY0_RSO6730, and MY0_RSO6735 were knocked out. The transformation was made by using DNA that contained the Erythromycin resistance cassette flanked by 1000 bp on each side. The sequence for the flanking regions and the genes can be found on NCBI Reference sequence NC_020286.1 for *Synechocystis sp.* PCC 6803.

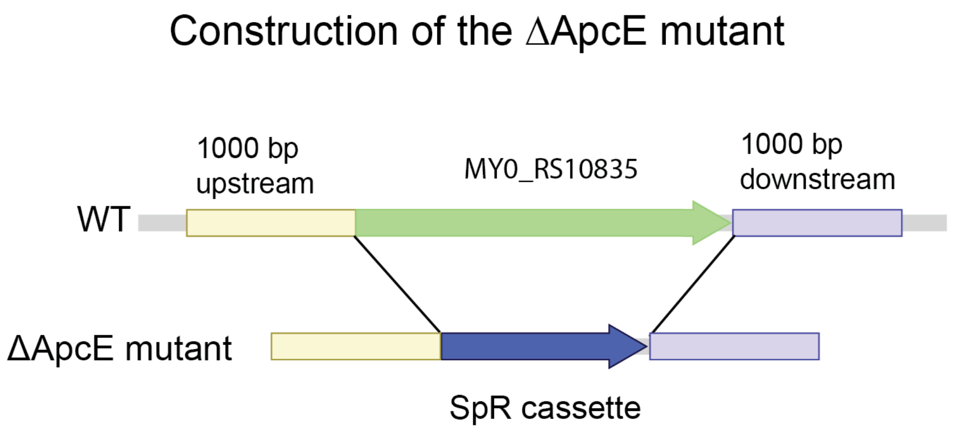

Fig. S4 Wildtype *Synechocystis sp.* PCC 6803 (WT) gene MY0_RS10835 is shown by a green arrow. The upstream and downstream regions of 1000 bp each is highlighted on either sides of the gene. The gene is knocked out in the ΔApcE mutant and a Spectinomycin resistance cassette (SpR) is inserted between the flanking regions.

Fig. S4 shows how the ApcAE deletion mutant was constructed. The gene MY0_RS10835 was knocked out. The transformation was made by using DNA that contained the Spectinomycin resistance cassette flanked by 1000 bp on each side. The sequence for the flanking regions and the gene can be found on NCBI Reference sequence NC_020286.1 for *Synechocystis sp.* PCC 6803.

Table S1 Primers for PCR confirmation of ΔCpcBD, ΔApcAC, ΔApcE, and PSI-kd.

Primer	Sequence 5' to 3'
CpcBD-F	CATAAAGTCAAGTAGGAGATTAATTCA
CpcBD-R	GCTCAAATAGTAATTAACAAAATAGC
ApcAC-F	CTTTACGGAGGAATCCATCC
ApcAC-R	GCAACAACTAGTCTCAAGC
ApcE-F	GTTAATGGGAGATAATGAAATCCA
ApcE-R	AGCTTTAAACAACCGCAA
PSI-kd-F	CCTTGCGGACTCTGAGCCAATTTGT
PSI-kd-R	CAGTGAAGGGGCCCTTGTGGGC
PsbH-F	ATTTAGTCATTTTTACGGGAAGTCT
PsbH-R	GAATATGTCAACAACCCCCCA

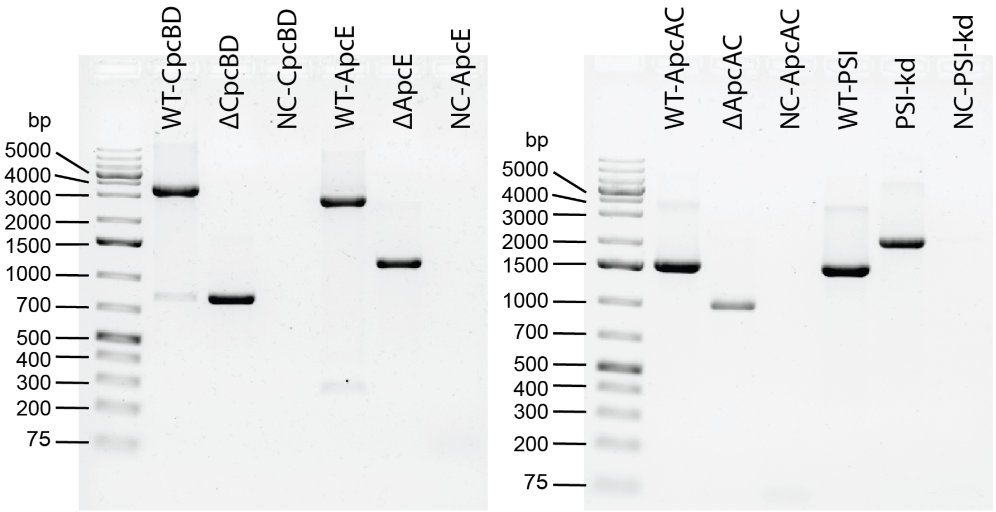

Fig. S5 PCR validation for transformation of ΔCpcBD, ΔApcAC, ΔApcE, and PSI knockdown in the PSIkd/ΔPBS strain.

Deletion mutants and PSI knockdown were confirmed by colony PCR and agarose gel electrophoresis (Fig. S5). PCR products were mixed with 6X TriTrack DNA Loading Dye (Thermo Fisher Scientific) and separated using a 1% agarose gel stained with SYBR™ Safe DNA Gel Stain (Thermo Fisher Scientific), using GeneRuler 1 kb Plus DNA Ladder (Thermo Fisher Scientific) as a size standard. PCR primers (Table S1) flanking each deletion region were used to amplify genomic DNA from wild-type and mutant colonies, with a no-template negative control included for each mutant.

For the phycocyanin deletion mutant (Fig. S5), the wild-type (WT-CpcBD) amplification produced a 3426 bp band comprised of the region specified in Fig. S2. The Δ*CpcBD* mutant (ΔCpcBD) carrying the Gentamicin resistance cassette yielded a smaller 804 bp product.

For the linker protein deletion mutant (Fig. S5), the wild-type (WT-ApcE) amplification produced a 2706 bp band comprised of the region specified in Fig. S4. The Δ*ApcE* mutant (ΔApcE) carrying the Spectinomycin resistance cassette yielded a 1161 bp product.

For the allophycocyanin deletion mutant (Fig. S5), the wild-type (WT-ApcAC) amplification produced a 1522 bp band comprised of the region specified in Fig. S3. The Δ*ApcAC* mutant (ΔApcAC) carrying the Erythromycin resistance cassette yielded a 987 bp product.

For the PSI knockdown (Fig. S5), the wild-type (WT-PSI) amplification produced a 1493 bp band comprised of the PSI promoter region and a part of the *psaA* gene. The PSI-kd mutant (PSI-kd) carrying the Chloramphenicol resistance cassette and the *PpetJ* promoter yielded a 2077 bp product.

No bands were observed in any of the negative controls (NC-CpcBD, NC-ApcE, NC-ApcAC, and NC-PSI-kd), confirming the absence of contamination (Fig. S5).

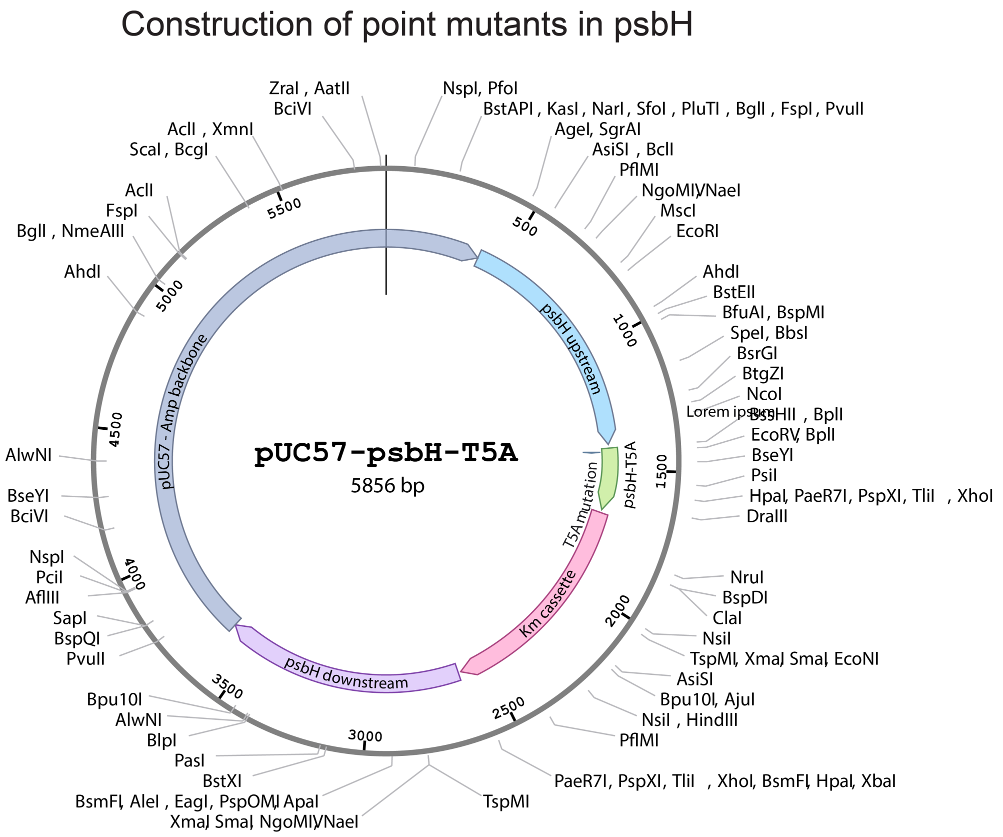

Fig. S6 Schematic map of plasmid pUC57-PsbH-T5A used for site-directed mutagenesis. Key features include a *psbH* upstream region (1000 bp), *psbH* gene, kanamycin resistance cassette (1032 bp), and *psbH* downstream region (1000 bp). The plasmid was constructed by Synbio Technologies.

The pUC57-psbH-T5A plasmid shown in Fig. S6, commercially synthesised by Synbio Technologies, has the Thr5 codon, ACT, changed to GCT. The change in codon for all Thr5 mutants is summarized in Table S2. DNA from all seven Thr5 mutants was amplified and sequenced for the inserted *psbH* gene using primers PsbH-F and PsbH-R (Table S1), which amplified a 1273 bp product spanning the *psbH* gene and the Kanamycin resistance region. The primers are located within the *psbH* upstream and downstream regions shown in Fig S6. Sequencing was performed by Plasmidsaurus, and the confirmed codon changes are summarized in Table S2. The remainder of the *psbH* gene was unchanged in all seven mutants, confirming the specificity of the mutagenesis strategy.

Table S2 **Point mutants T5A, T5D, T5E, T5H, T5K, T5R, and T5S in the PSIkd/ΔPBS background were sequenced and the confirmation of the mutation is shown in the table.**

Mutation	Expected Codon	Sequencing result
T5A	GCT	GCT
T5D	GAT	GAT
T5E	GAA	GAA
T5H	CAT	CAT
T5K	AAA	AAA
T5R	AGA	AGA
T5S	TCT	TCT

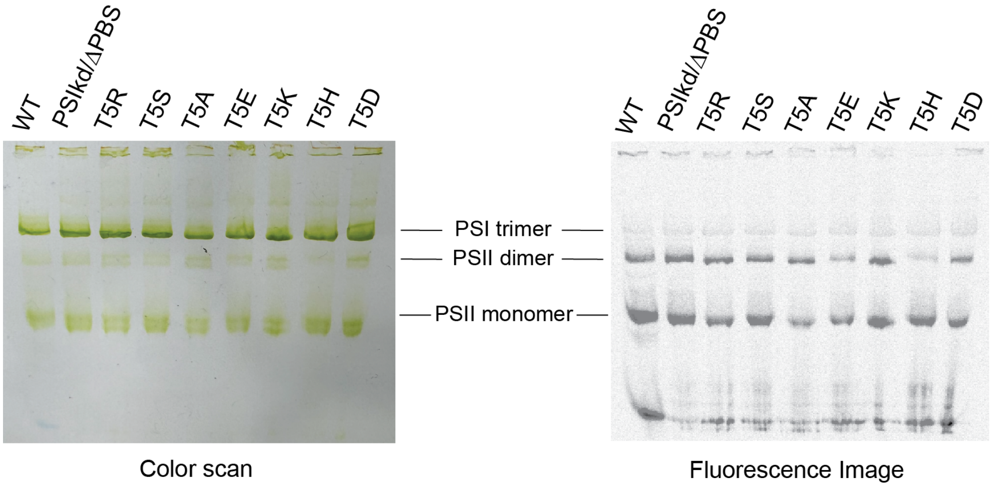

Fig. S7 Clear-native PAGE images: Color scan on the left and fluorescence image on the right comparing WT, PSI-kd/ΔPBS and the point mutants T5R, T5S, T5A, T5E, T5K, T5H, and T5D in the PSI-kd/ΔPBS background. WT, PSI-kd/ΔPBS and the point mutants T5R, T5S, T5A, T5K, and T5D show comparable PSII dimer intensities. T5E, on the other hand, shows a weak PSII dimer, and T5H shows the weakest band of all.
